# A System-of-Systems Bio-Inspired Design Process: Conceptual Design and Physical Prototype of a Reconfigurable Robot Capable of Multi-Modal Locomotion

**DOI:** 10.3389/fnbot.2019.00078

**Published:** 2019-09-20

**Authors:** Ning Tan, Zhenglong Sun, Rajesh Elara Mohan, Nishann Brahmananthan, Srinivasan Venkataraman, Ricardo Sosa, Kristin Wood

**Affiliations:** ^1^Key Laboratory of Machine Intelligence and Advanced Computing, Ministry of Education, School of Data and Computer Science, Sun Yat-sen University, Guangzhou, China; ^2^School of Science and Engineering, Chinese University of Hong Kong, Shenzhen, China; ^3^Shenzhen Institute of Artificial Intelligence and Robotics for Society, Shenzhen, China; ^4^Engineering Products Development Pillar, Singapore University of Technology and Design, Singapore, Singapore; ^5^Department of Design, Indian Institute of Technology Delhi, New Delhi, India; ^6^Art Design & Architecture, Monash University, Melbourne, VIC, Australia

**Keywords:** bio-inspired design, system-of-systems, multi-model locomotion, reconfigurable robots, mobile robotics

## Abstract

Modern engineering problems require solutions with multiple functionalities in order to meet their practical needs to handle a variety of applications in different scenarios. Conventional design paradigms for single design purpose may not be able to satisfy this requirement efficiently. This paper proposes a novel system-of-systems bio-inspired design method framed in a solution-driven bio-inspired design paradigm. The whole design process consists of eight steps, that is, (1) biological solutions identification, (2) biological solutions definition/champion biological solutions, (3) principle extraction from each champion biological solution, (4) merging of extracted principles, (5) solution reframing, (6) problem search, (7) problem definition, and (8) principles application & implementation. The steps are elaborated and a case study of reconfigurable robots is presented following these eight steps. The design originates from the multimodal locomotion capabilities of two species (i.e., spiders and primates) and is analyzed based on the Pugh analysis. The resulting robotic platform could be potentially used for urban patrolling purposes.

## Introduction

A design process is a systematic approach followed by designers while trying to solve a problem, which could be as simple as designing a chair or could be as complex as designing an aircraft (Mas et al., 2012). Irrespective of the complexity of the problem, looking for inspiration before starting to design has been a normative step in the world of design (Eckert and Stacey, 2000). Especially when it comes to exploration and discovery of new ideas (Murakami and Nakajima, 1997). Therefore, designers have started to follow a systematic approach to seek inspirations outside the problem domain to find solutions (i.e., domain-independent) where the problems are closely related to the original problem domain (López-Mesa, 2011).

The bio-inspired engineering design approach is one of systematic approaches using analogies from biological creatures in the nature to develop solutions for handling engineering problems (Helms et al., 2009; Eroglu et al., 2011a). There are many practical examples, such as the invention of Velcro (Versos and Coelho, 2011) and conceptual design of the Bionic Car project (Vincent and Man, 2002; Floyd et al., 2006). This also applies to the scientific world, for example, the nano-scale superhydrophobic coatings inspired by the self-cleaning mechanism of lotus leaves (Cheng and Rodak, 2005), the imitation of the pinecones to design clothes that can regulate body temperature (Groeneveld, 2008), and the design of micro-robots that can walk on water, mimicking the locomotion of the basilisk lizard (Zari, 2007).

Modern engineering problems often require solutions with intrinsic compliance, for better function variety, environment adaptivity, and structure flexibility. As a result, soft materials start to emerge in the bio-inspired robotics design, such as the traditional cable-driven mechanism, spring-damper structure, and recent pneumatic artificial muscles. But the intrinsic drawback of using these materials is their lacking in the accuracy and reliability, making the design loss in robustness. As an alternative solution, reconfigurable design in robot becomes appealing, where not only it inherits the accuracy and robustness of rigid structure, but also it is able to achieve the compliance as desired using reconfiguration. In such a case, the intrinsic compliance roots in the reconfigurability, instead of the materials and flexible structures.

However, a systematic design methodology for such bio-inspired design is still missing, making the process deducing challenging (French, 1994; Benyus, 1997). There are two main different approaches with respect to different starting points, that is, the Problem-Based Bio-Inspired Design (PB-BID) process and the Solution-Based Bio-Inspired Design (SB-BID) process (Eroglu et al., 2011b). In order to include a wide variety of functionalities at the beginning of the design process, here we stick to the SB-BID process since it is more appropriate to implement the system-of-systems paradigm. There are a few methods available for the SB-BID process where most of prominent models belong to the Aalbog's method (Colombo, 2007) and Helms' method (Helms et al., 2009). In general, both Aalbog's and Helms' models begin with the identification of biological solutions, and then extract principles from the identified solution. The latter method involves the reframing and application of extracted principles to solve a real-world engineering problem. However, these previous models mainly led to creating a solution inspired by a single biological species for solving a single problem, with poor extensibility.

In this paper, we presented a novel BID process to creating engineering product with multiple functionalities. The inspiration is originated from multiple biological species to solve multiple problems. Since the process involves multiple biological systems (i.e., species), we name it as the System-of-Systems Bio-Inspired Design (SoS-BID) process. The process is firstly introduced and a case study will be demonstrated followed this design process, for designing a reconfigurable robot called Scorpio, which is able to change its morphologies to achieve different locomotion modes, namely crawling, rolling, and wall-climbing.

## System-of-Systems Bio-Inspired Engineering Design Process

Typically, a design process would be dynamic, which changes frequently based on the context of the problem faced and the available solution space (Vattam et al., 2007). Thus, the process undergoes repetitive reformulations of both design problems and solutions. In the following sections, we present the proposed SoS-BID process to solve the design problem of interest. The process is a systematic approach, which includes the following eight steps:

- Step 1: Biological solutions identification.
- Step 2: Biological solutions definition/Champion biological solutions.
- Step 3: Principle extraction from each champion biological solution.
- Step 4: Merging of extracted principles.
- Step 5: Solution reframing.
- Step 6: Problem search.
- Step 7: Problem definition.
- Step 8: Principles application & implementation.

**Figure 1:**
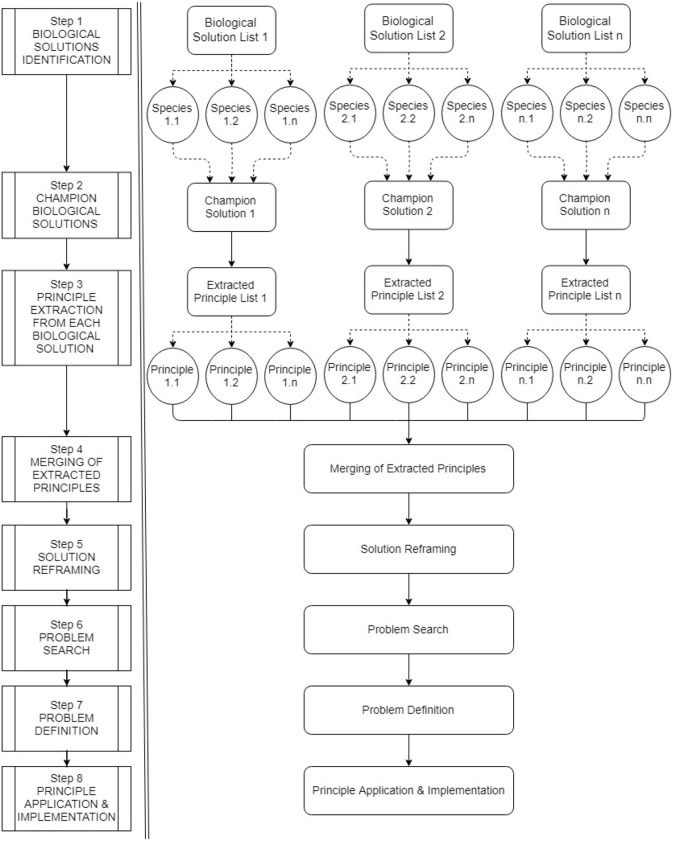
System-of-systems bio-inspired design process (systematic diagram).

A systematic diagram, explaining an overview of the proposed SoS-BID process framework is shown in Figure 1.

### Step 1: Biological Solutions Identification

In the very first step of the SoS-BID process, designers start with multiple biological solutions of interest as inspiration sources that will potentially be used to solve a certain problem (Lindemann and Gramann, 2004). This step involves the search of biological species which perform desired biologized tasks. Instead of randomly observing, here we propose to observe the biological species based on the taxonomy which is the science of defining biological species based on shared characteristics. The list of species could be categorized according to their specific abilities or nature (Huxley, 1875; Shane et al., 1986; Santori et al., 2005; Alexander, 2006; Manohar et al., 2016) as follows:

- Based on locomotion modes (e.g., crawling, swimming, flying, and jumping).
- Based on appearances (e.g., size and color).
- Based on living conditions and mediums (e.g., terrestrial, arboreal, and aquatic).
- Based on scientific classifications (e.g., kingdom, class, and family).
- Based on social organization patterns (e.g., solitary and social).

Once the categories are defined, the biological solution search is conducted under each specifically defined category. The output of this step would be a series of categories and a list of biological species for each category.

### Step 2: Biological Solutions Definition/Champion Biological Solutions

It is noted that the selection from the biological solutions identified in Step 1 could be subjective. However, we have defined a few general selection criteria of the species to assist the designers in decision making, such as the task performance efficiency, multi-functional capability, ease of principle extraction, ease of kinematic study, and practical feasibility. At this step, the following existing design-concept-selection methods can be adopted for analysis:

- Pugh's Concept Selection (Muller et al., 2011).
- Weighted Rating Method (Ulrich and Eppinger, 1995).
- Analytical Hierarchy Process (Saaty, 2013).
- Electre Method (Roy, 1991).

The selected species is called the champion biological solution/species. The output of this step would result in a single champion biological species selected for each category.

### Step 3: Principle Extraction From Each Champion Biological Solution

This step involves a deeper understanding of the selected biological species regarding their functions and behavior, and identification of the underlying principles used to solve a problem in the context of nature. Finally, through the analysis of the species, the important fundamental principles are extracted from the champion biological species.

### Step 4: Merging of Extracted Principles

The extracted principles are analyzed for identifying repetitive principles. This step involves the removal of repetitive principles and merging of the resulting principles into a series of unique abstract principles (functions, behaviors, etc.) in general.

### Step 5: Solution Reframing

This step is to transfer the solution from the biological domain into the engineering domain. In particular, the reframing step forces the designers to think how humans might view the significance of the biological principles extracted. The output of this process would result in a series of useful applications and related constraints of the biological principles in the human-society context, which are labeled as “humanized solutions.”

### Step 6: Problem Search

Given the generated humanized solution and related constraints, this step is to find a potential engineering problem that the solution could be applied to. The problem could be an existing one or an entirely newly defined problem.

### Step 7: Problem Definition

The problem definition plays an important role in the SoS-BID process, involving deep and higher level understanding and interpretation of the searched problem. Generally, this step includes the following sub-steps:

- Generating preliminary design criteria.
- Evaluating intermediate engineering reasonings.
- Generating system requirements.
- Merging of system requirements if needed.

It is worth noting that the resultant criteria generated above may still be abstract, based on which the specific and detailed system requirements are to be derived.

### Step 8: Principles Application & Implementation

This step involves the real transformation and implementation of the principles identified in the biological domain into the engineering domain. In other words, the biological-solution-applicable principles have been translated into engineering terminologies to facilitate the process of implementation. The output of this step results in an embodied principle of the engineering concept that satisfies the need of one or several practical engineering problems as defined in the previous step.

To capture the general features of the SoS-BID process, a summary of the full process is presented in Table 1 with some short descriptions.

**Table 1:** A summary of the system-of-systems bio-inspired design process.

**Steps**	**Description**
Biological solutions identification	Observation, identification, and categorization of a few interesting biological species and record solutions of interest **Output**: a series of categories and a list of biological species for each category
Biological solutions definition/Champion biological solutions	Understanding the biologized problem that each biological solution is solving and selecting a single/champion biological solution for each biologized problem space (biologized task) **Output**: a single champion biological species selected for each category
Principle extraction from each champion biological solution	Principle extraction for champion species regarding their functions and behavior **Output**: a series of solution-applicable principles for each category (biologized category)
Merging of extracted principles	Removal of repetitive principles and mergence of resulting principles **Output**: merged principles
Reframe the solutions	Reframing the solution and applicable principles in a context useful to human engineers **Output**: reframed humanized solutions
Problem search	Searching or defining the solution-applicable problem which could be existing problems or entirely new problems. **Output**: solution-applicable problems
Problem definition	Higher-level understanding and interpretation of the searched problem and identification of design criteria; Derivation of detailed system requirements **Output**: system requirements
Principle application & implementation	Translation and implementation of the principle into the searched or defined problem **Output**: real applications

## Development of the Scorpio Robot Using the Proposed System-of-Systems Bio-Inspired Design Process

### Step 1: Biological Solutions Identification

To begin the SoS BID process, we decide to look at the species with shared characteristics of their locomotion capabilities. The locomotion aspect of biological species refers to that how biological species in nature maneuver from one point to another. Here the locomotion modes are categorized into two major categories of interests:

- Species which can perform planar locomotion.
- Species which can perform vertical locomotion.

In such manner, the locomotion in space can be seen as the combination of these two types of locomotion. Based on the two categories, the biological species are identified using academic articles from Google scholar and non-academic articles from other online search engines, and categorized as follows.

#### Category 1: Species Which Can Perform Planar Locomotion

It is found that most of the species are capable of performing multiple locomotion modes (Jenkins, 2012; Lock et al., 2013). Especially, they perform different locomotion modes based on the encountered different scenarios (Kuroda et al., 2014). Some are even capable of changing their shapes in order to perform multiple locomotion gaits (Prostak, 2014; Nemoto et al., 2015; Mintchev and Floreano, 2016; Grzimek's Animal Life Encyclopedia, 2017). Species in nature such as snakes, lizards, and insects can adapt their gaits in response to different types of terrains that they navigate, which vary from smooth terrains to bumpy ones (Bagheri et al., 2015). Most species switch their locomotion modes to traverse different terrains, for example, to overcome obstacles such as pits and bumpy surfaces on their ways. Hereby, we select five species in nature, which can change their shape to perform multiple locomotion modes, for further analysis.

- Mount Lyell Salamander

Salamanders generate tetrapod postures to help them to walk (Cabelguen et al., 2010). A larger portion of its energy is used up for lifting their body for walking which results in a slower motion. Most salamanders found in nature can partially curl up their body, tails, and legs as a defense mechanism and limits them from performing rolling locomotion. The Mount Lyell salamander is a species found in the northern Sierra Nevada in California, which can curl up its entire body to form a spherical shape (Mintchev and Floreano, 2016). Such a spherical morphology enables it to roll down a slope without getting injured (King, 2013).

- Woodlouse

Woodlouse is an isopod species which belongs to the Armadillidae family which normally walks most of the time. It is also capable of rolling up its entire body to form a spherical shape as a defense mechanism and to roll down a slope without getting injured (Grzimek's Animal Life Encyclopedia, 2017).

- Moth Caterpillar

Moth caterpillar belongs to the Lepidoptera family which is also capable of walking and rolling. While being poked, it will perform a backward roll by curling up into a ball shape. The curling movement triggers a momentum for rolling locomotion. Once it depleted the momentum, it needs to relax and trigger a new curling movement to perform the rolling locomotion once again (King, 2013).

- Wheel Spider

The wheel spider is a kind of huntsman spider found in Namib desert. The spider possesses two types of locomotion modality, i.e., crawling and rolling (Nemoto et al., 2015). While entering a slope, it flips sideways and runs cartwheel down the slope, which can produce around 44 turns per second (Leroy and Leroy, 2003).

- Cebrennus rechenbergi

**Figure 2:**
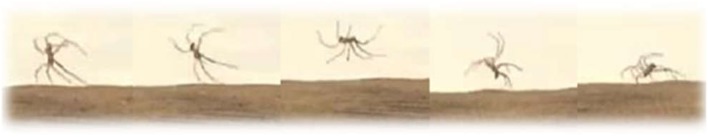
The rolling sequence generated by *Cebrennus rechenbergi*.

*Cebrennus rechenbergi* is found in Morocco deserts, belongs to the family of huntsman spider, which is also known as the Moroccan flicflac spider. Like the species mentioned above, such a kind of spider is also capable of two types of locomotion modality, namely crawling and rolling. It is also known uniquely for its rolling locomotion within the spiders' family. Once being threatened, it can multiply its speed by performing the acrobatic flicflac somersault locomotion (Figure 2) which assists to propel off the ground to go uphill, downhill, and on flat terrain (Prostak, 2014).

Table 2 showcases the different locomotion modes performed by the species in Category 1.

**Table 2:** Locomotion performed by five species of Category 1.

**Species**	**Locomotion type 1**	**Locomotion type 2**
Mount Lyell salamander	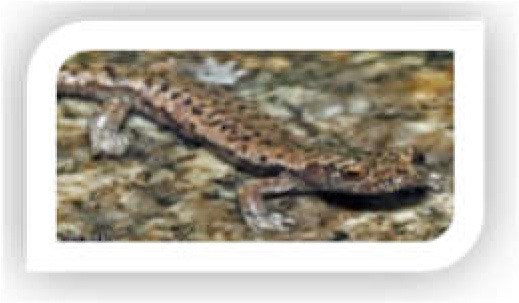 Walking	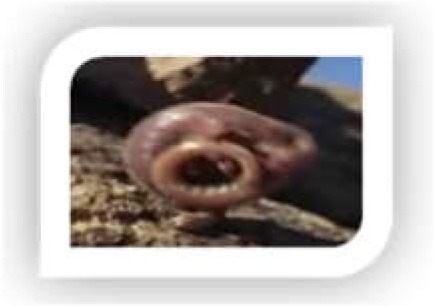 Passive rolling down a slope
Woodlouse	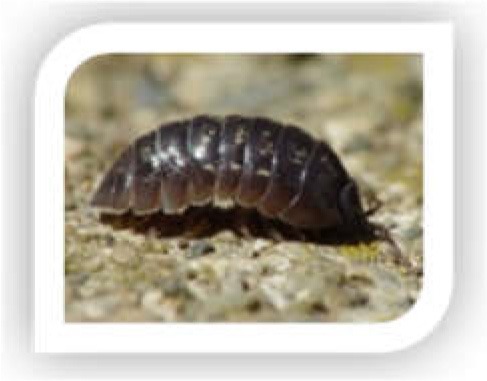 Walking	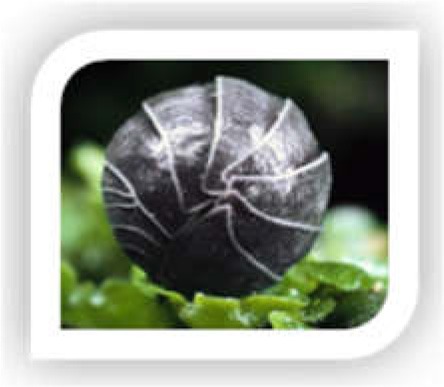 Passive rolling down a slope
Moth caterpillar	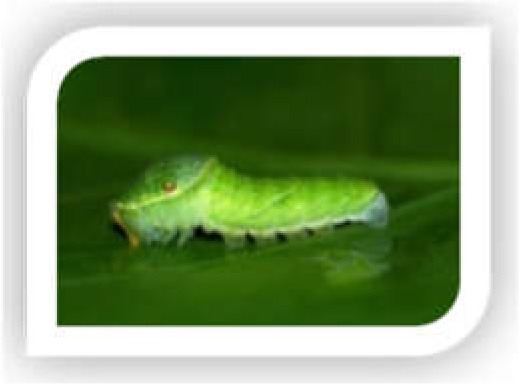 Walking	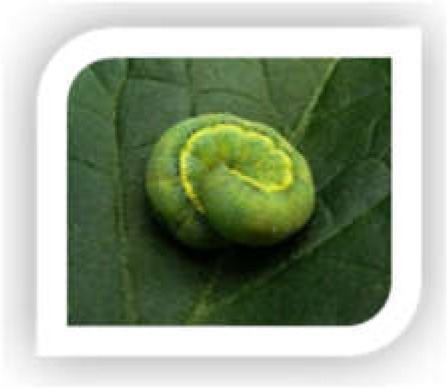 Active backward rolling
Wheel spider	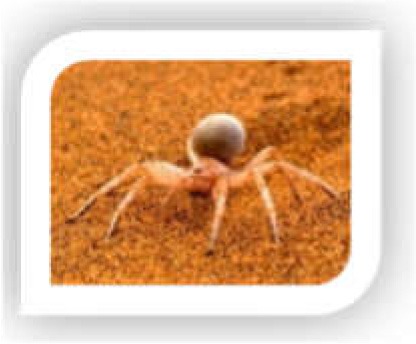 Crawling	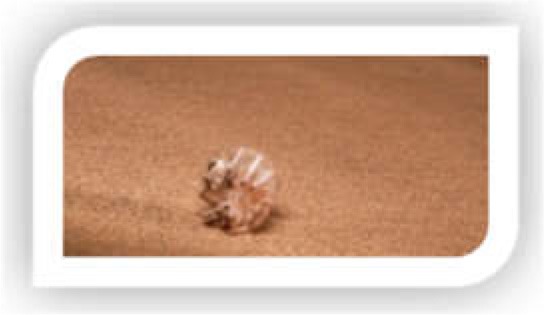 Cartwheeling down a slope
*Cebrennus rechenbergi*	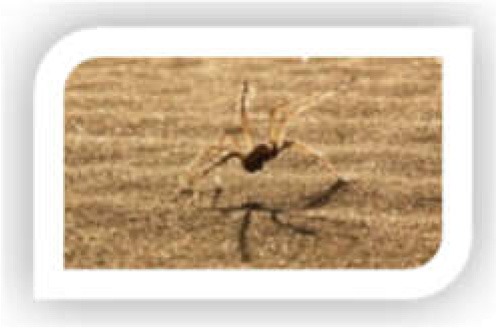 Crawling	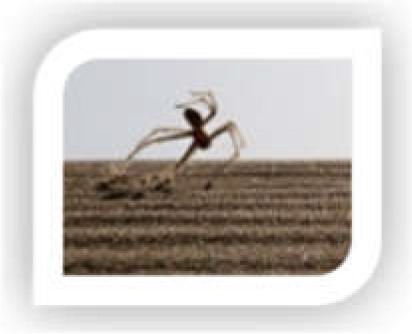 Active flicflac somersault

#### Category 2: Species Which Can Perform Vertical Locomotion

The planar movement on the flat land is much easier for animals compared to climbing on vertical and inclined surfaces. One of the reasons is because the planar movement does not require much work against gravity (Kissling, 2004). Most climbing species have a unique adaptation to their climbing locomotion in nature. In general, all of them have strong grasping capabilities (Gebo and Dagosto, 1988) and locomotion mechanisms that enable them to keep their body's centers of gravity as close as possible to the object climbed (Clark et al., 2007). Based on the biological solution searching, we briefly analyzed the locomotion patterns of five different climbing species in nature as follows:

- Spider

Like many species, spiders possess the ability to walk and climb. Most spiders' legs consist of microscopic hair, enabling them to stick to the wall based on the electrostatic attraction, such as van der Waals force (Spolenak et al., 2005).

- Snake

Snakes use certain modes of locomotion to move. For example, they use friction to climb steeper surfaces where part of the body has a grip on the surface of interest climbed and the other part extends forward. The alternating between contracting and extending of their body in such a way assists them in climbing and descending a wall (Marvi and Hu, 2012).

- Gecko

Gecko's feet process a lot of microscopic tiny bristles called setae. Similar to the spider, these setae can leverage the Van der Waals force to stick to the wall (Sitti and Fearing, 2003).

- Primate

Primates consist of hands, feet, opposable thumbs, and big toes. They have broader fingertips with nails which can apply great gripping and grasping strength to objects. These features enable them to perform climbing (i.e., vertical clinching and leaping) locomotion. On the other hand, they have other specialized modes of locomotion other than climbing, which captured our attention the most such as below-branch and knuckle-walking locomotion (Jenkins, 2012).

- Snail

Snails move their bodies by gliding through a mucus layer secreted by one of its glands. This layer combined with its smooth flat base enables them to climb walls by creating a strong suction (Chan et al., 2005).

Table 3 below describes the different locomotion modes performed by the species of Category 2.

**Table 3:** Locomotion performed by five specie of Category 2.

**Species**	**Locomotion type 1**	**Locomotion type 2**
Spider	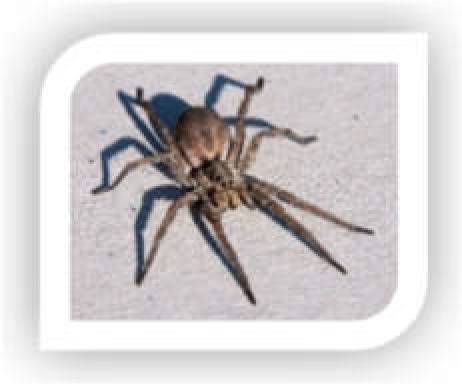 Walking	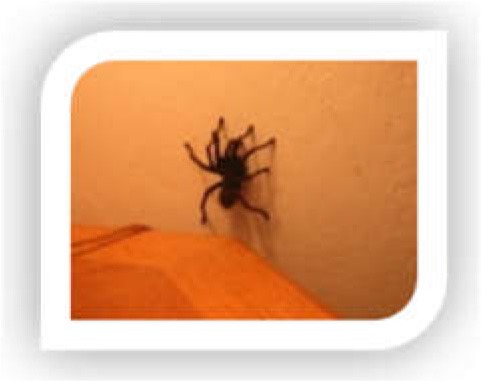 Climbing
Snake	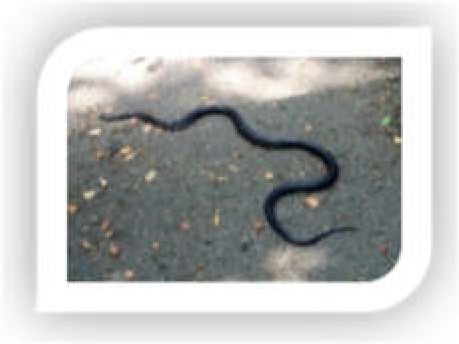 Walking	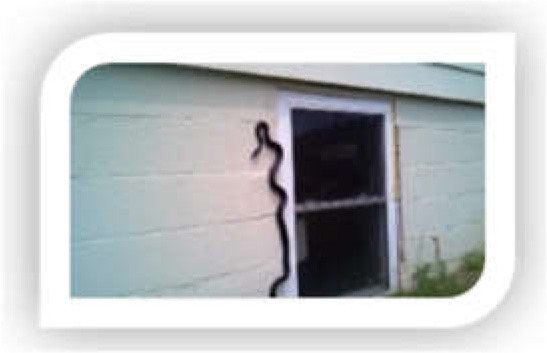 Climbing
Gecko	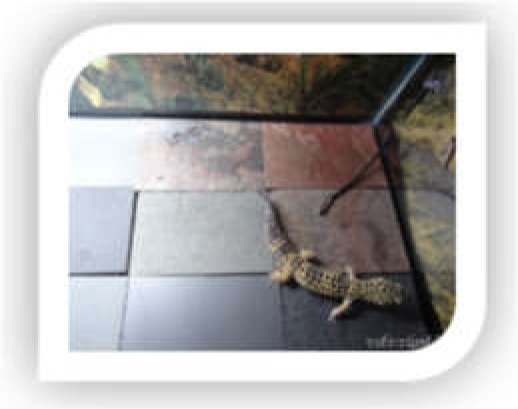 Walking	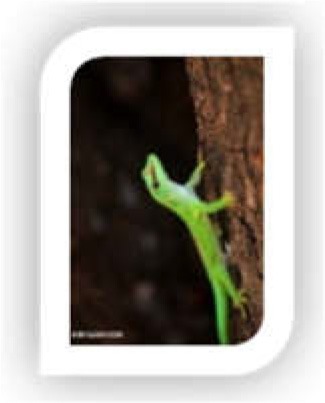 Climbing
Primate	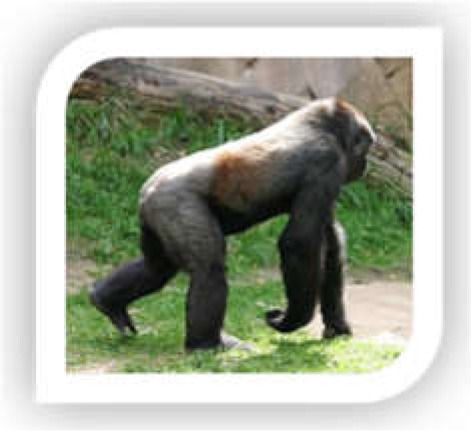 Knuckle walking	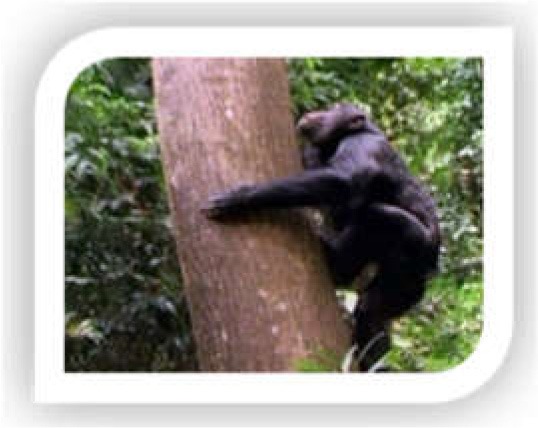 Climbing (Clinching and Leaping)
Snail	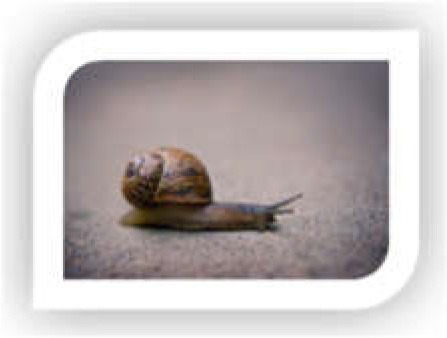 Sliding walk	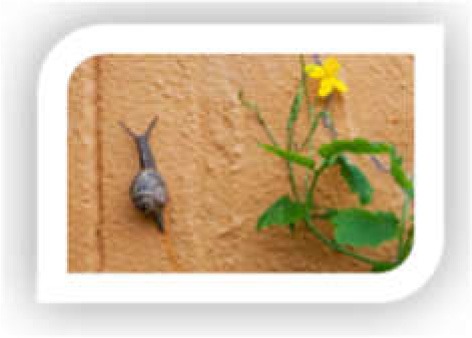 Sliding climb

### Step 2: Biological Solutions Definition/Champion Biological Solutions

The selection of the champion biological solution for each category is identified in this step. Each biological species is ranked based on a few key criteria for selection. For Category 1, we choose woodlouse as the benchmark option and evaluate the rest of the candidates against woodlouse for each key criterion. The key criteria for Category 1 are listed below:

- The ability to travel fast in land terrains using rolling locomotion.
- The ability to perform active rolling locomotion.
- The ability to navigate is varied speed.
- The ability to overcome obstacles.
- The ability to perform multifunctional task performance capability.
- The ability to change its heading direction based on its own will.
- The ability to perform rolling locomotion with minimal rest.
- The stability of performing crawling and walking locomotion.
- The stability of performing rolling locomotion.

For Category 2, we choose the snail as the benchmark option and evaluate the rest of the alternatives against the snail for each key criterion. The key criteria for Category 2 are as follows:

- Proof of concept to overcome the gravitational pull.
- Specialized mechanical movements.
- The ease in replicating the biological principle into a working prototype or product.
- The ability to navigate in varied speed.

**Figure 3:**
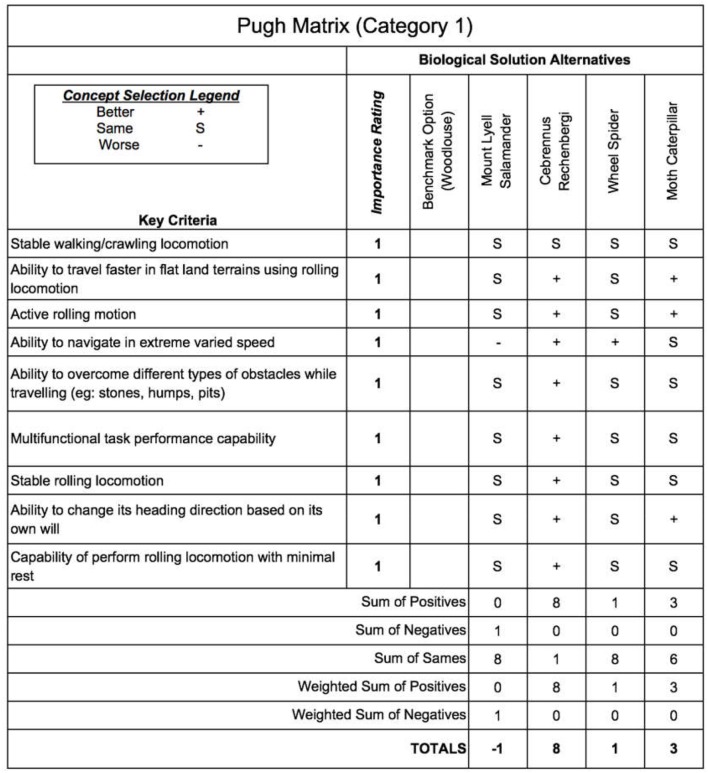
The Pugh analysis for Category 1.

**Figure 4:**
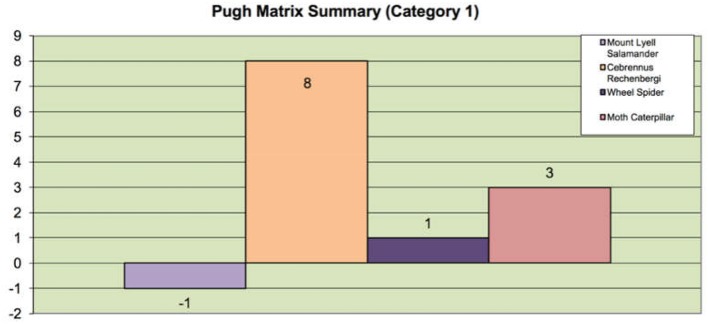
The Pugh summary for Category 1.

**Figure 5:**
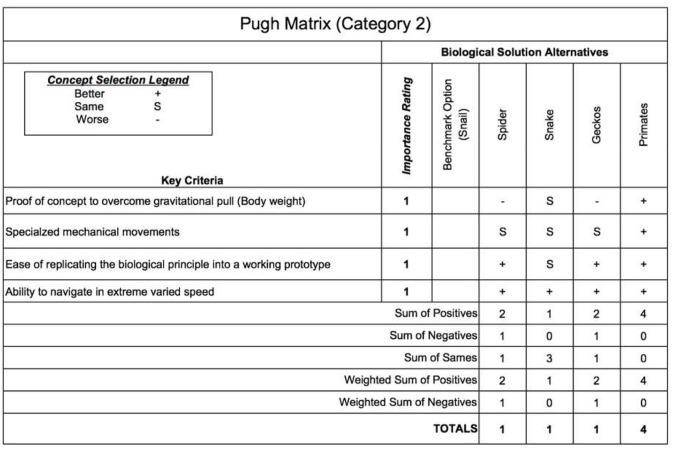
The Pugh analysis for Category 2.

**Figure 6:**
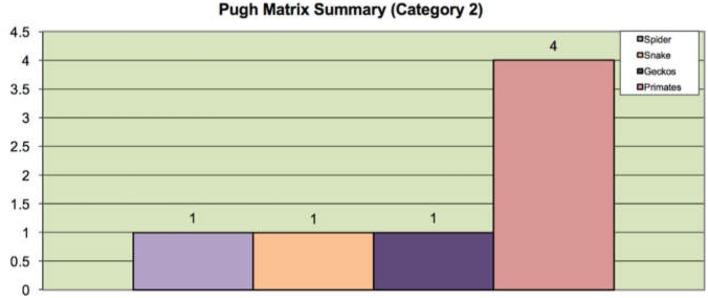
The Pugh summary for Category 2.

These criteria are selected features of the species. In this step of the design process, we ought to choose one champion biological species for each category from the list of biological species. Different selection methods could be used for the selection process. In this paper, the Pugh analysis is used to evaluate the candidate species against a baseline species (benchmark option) to select the champion species. Pugh analysis is a decision-making model used when a choice has to be made given a list of candidates (Muller et al., 2011). These candidates are compared based on the selection criteria, which are designed and summarized in accordance with the need of the context. As shown in Figures 3, 5, we use three concept selection legends, i.e., “+”, “–”, and “S” where the symbol “+” means that the candidate species is better than the baseline species'; “–” denotes that the candidate is worse than the baseline species; “S” represents that the candidate is the same as the baseline species. Each key criterion can be given a weight, also known as importance rating. In our case, equal importance weight was used to all the key criteria. Once the scores are assigned to each species, the sum and weighted sum are calculated, and then the candidate with the highest positive score is selected as the champion species. If all the final total scores are in negative, then the baseline species is selected as the champion species. Finally, based on the Pugh analysis illustrated in Figures 4, 6, “*Cebrennus rechenbergi*” and “Primate” are selected as the champion species for Category 1 and 2 correspondingly.

It is worth mentioning that the Pugh analysis used here is for the purpose of comparing objectively different species, which is simple and efficient. Of course, Pugh analysis is not always the best method, but it works and is compatible with the proposed framework. Other methods can probably also work and are compatible, and may be better than Pugh analysis in some cases. Our framework is open and flexible to integrate any candidate selection methods, because the selection step is not decided by other steps. Actually, there is very little study about the general bio-inspired design process. Instead, most of bio-inspired studies are related to the development of a specific robot. Therefore, as far as we know, Pugh analysis is the first one and there is no other available on selection methods, especially for such a new bio-inspired design framework.

### Step 3: Principle Extraction From Each Champion Biological Solution

The functions and behavior of the species concerning solving problems in the context of nature are as follows.

- Category 1: *Cebrennus rechenbergi*
- Like all the spiders, *Cebrennus rechenbergi* can locomote in crawling mode using its eight legs until an external stimulus provokes and disrupt its motion.
- Once threatened, it can double its speed by switching from crawling to the forward or backward acrobatic flicflac somersault movement. Ingo Rechenberg from TU Berlin discovered this fascinating behavior (King, 2013; Prostak, 2014).
- Category 2: Primates (Apes)

All the primates are natural climbers where some of them possess specialized attributes to perform other types of locomotion such as vertical clinching locomotion and knuckle-walking/jumping locomotion. Identification of underlying scientific principles in the functions and corresponding behaviors of the species are as follows:

- Category 1: *Cebrennus rechenbergi*
  - The acrobatic flicflac somersault movement is an active rolling locomotion where it does not require any additional force from the surrounding such as the gravitational pull of the earth.
  - The species does not require any additional initiation gestures such as crawling forward to generate the rolling locomotion.
  - Existing research proves that the species forms an abstract wheel with its legs and rotates its whole body to perform the rolling locomotion (King, 2013).
- Category 2: Primates (Apes)
  - While performing vertical clinching, the species tends to keep the center of mass closer to the object it is climbing, which in return mitigates the energetic expenditure during climbing (Jenkins, 2012).
  - Knuckle-walking is a form of quadrupedal walking which is performed by gorillas and chimpanzees. When they walk forward, the whole-body weight is held on to their knuckles. When they are threatened, they perform an instant long jumping locomotion, where both their knuckles are struck onto the ground together while simultaneously pushing forward its body to the direction of motion.

The extracted principles from the locomotion patterns of the species are as follows:

- Category 1: *Cebrennus rechenbergi*
  - The spider uses eight legs for crawling locomotion.
  - The spider uses four legs for rolling locomotion.
  - The spider forms a pseudo wheel using its half spherical shaped legs while rolling.
  - d) The spider performs active rolling.
- Category 2: Primates (Apes)
  - The primate keeps the center of mass close to the object of interest being climbed to mitigate energetic expenditure.
  - The primate (like gorillas and chimpanzees) performs knuckle walking locomotion.

### Step 4: Merging of Extracted Principles

The previous step extracts the principles of two species, respectively, but does not merge them. Here the following abstract principles can be refined based on the two sets of principles:

- The species uses eight legs for crawling.
- The species uses four legs for and rolling.
- The species forms a pseudo wheel using its half-spherical-shaped legs while rolling.
- The species performs active rolling.
- The species keeps the center of mass closer to the object of interest being climbed to mitigate energetic expenditure.
- The species can perform knuckle-walking locomotion.

Merging these different biological principles is our main contribution that these principles should be reflected in a single platform. In this case study, the merging of spider and primate's locomotive principles is specifically carried out by self-reconfiguration, through which the different locomotive gaits switch between each other.

### Step 5: Solution Reframing

The above-mentioned principles are analyzed once again for solving engineering problems in real applications. Then the corresponding solutions can be obtained after reframing as follows.

- The spider uses eight legs for its crawling which helps to distribute its body weight to all eight legs equally. In other words, a greater number of legs helps for a more stable crawling locomotion.
- Out of the eight legs, the spider uses only 4 of its legs to perform the somersault. In other words, in order to perform a task, it is not necessary to make use of all the available resources. The resources have to be precisely optimized based on the required need.
- The spider creates a half spherical shaped wheel with each of its leg. When two half spherical shaped wheels are put together, it creates a circular wheel which supports for a rolling locomotion. In other words, as an engineering designer, it is possible to create a vehicle or an artifact which can walk as well as roll by being able to reconfigure its physical structure.
- The spider performs active rolling when on flat terrains especially when threatened by an external factor. But while entering down into an inclined slope, it shifts to passive rolling so that it does not lose its balance on slope. The same concept can be adopted in engineering design in deciding on which scenario active and passive motion should be used.
- The spider rotates its whole body because the rotation causes a shift of its center of mass. This repeated shift in its center of mass supports its rolling locomotion. The same mechanism can be adopted in engineering design where instead of having side wheels to drive a vehicle, we can roll the vehicle in order for it to move from one point to another.
- The primate keeps the body close to the tree being climbed. This helps to keep the center of gravity close to the object of interest, reducing the amount of energy used for climbing. Likewise, the same concept could be adopted in the mechanism design for performing vertical locomotion. This in return reduces the overall energy used throughout the wall climbing locomotion and increases the battery lifetime of the artifact.

### Step 6: Problem Search

Once the merged solutions are reframed, we realized that most of our observations are more inclined toward locomotion and maneuverability on different types of terrains. The response of the species would be closely related to the terrain conditions. Therefore, one of the potential applications could be the surveillance task in unstructured environments, such as exploration of the urban terrorism. Over the past two decades, deaths due to terrorism have increased dramatically since year 2000. The urban terrorism is probably becoming the new norm where government authorities are taking prevention actions and coming up with new innovative ideas to strengthen their security systems (Muggah, 2016). The reconnaissance and surveillance tasks bring a huge life risk to ground soldiers, especially when it comes to urban search and rescue missions where soldiers need to patrol into buildings and area surroundings where there is no availability of prior information regarding the activists. Therefore, Intelligence Surveillance and Reconnaissance (ISR) systems are taking prominence. Recently the use of unmanned vehicles such as iRobot PackBot (McPherson, 2011), Roboteam MTGR (Steigerwald, 2015), Roboteam IRIS (ROBOTEAM, 2012), and CP-ISR nano drones (Menon, 2016) are being used to replace human soldiers. These robots are sent to the field to collect information using onboard sensors. However, their navigation and survival capabilities are restricted due to the visibility, noise, and types of terrains that they maneuver.

Thus, the aforementioned reframed solutions could be applied in the context of urban patrolling as the missions in this field are very unpredictable and require different types of behavior in order to execute the task. On the other hand, hiring, and retaining professional security personnel have become a major challenge for such missions. Therefore, the problem shifts to designing an unmanned ISR platform to be sent into a multistory building or space where it could silently navigate on multiple types of terrains, overcome obstacles, and survive from the eyes of the hostile force.

### Step 7: Problem Definition

Based on the problem scope, five preliminary design criteria are identified and presented as follows:

- A platform which could navigate through a multistory building.
- A platform which could navigate through multi-type terrains.
- A platform which could navigate freely through overcoming or avoiding obstacles.
- A platform which could survive from the eyes of the hostile forces.
- A platform which could maneuver silently.

The above-mentioned design criteria undergo an intermediate engineering reasoning step for further analysis, based on which the system requirements for the robot are generated. The requirements are a set of specifications of the robotic platform to behave in a certain fashion and will be used as the foundation in the principle application and implementation phase. The summary of the preliminary design criteria, engineering reasonings and system requirements for the robot are presented below:

- A platform can achieve silent motion through its capability to maneuver in slow motion.
- Most robots are unable to navigate through multi-type terrains due to their fixed morphologies. A wheeled robot can move freely on smooth and obstacle-free terrains based on its wheeled mobility, but the motion becomes highly restricted in marsh and tundra terrains like in a forest area. In contrast, legged robots can move freely in such terrains compared to wheeled robots, but their motion is not efficient enough on smooth terrains due to its speed. Therefore, designing a platform capable of changing its morphology through reconfiguration would be able to solve the problem of maneuvering through multi-type terrains.
- Most fixed morphology robots motion becomes restricted when they encounter obstacles such as hump and get cornered into a position where it cannot recover itself. Therefore, designing a platform which can reconfigure itself by changing its morphology would be able to solve the problem of overcoming a series of obstacles during its motion.
- For a robot to survive from the eyes of the activists, it is a compulsory requirement for the robot to be smaller.
- A robot can achieve multistory navigation in an indoor setting only by flying, staircase climbing, and wall climbing. Flying would create more visibility and could eventually alert the activists. Staircase climbing is challenging due to the miniaturized version of the robot. In other words, the step size of the staircase would restrict the robot's motion which makes wall climbing as the only choice for navigation through a multistory building. Therefore, designing a platform which can wall climb would be able to solve the problem of multistory navigation.

The following system requirements are generated based on these reasonings.

- SR1: Design a robot platform capable of overcoming obstacles through its ability to reconfigure its morphology and perform multiple modes of locomotion in multi type terrains.
- SR2: Design a robot platform which can perform wall climbing.
- SR3: Design a robot which can navigate at variable speeds.
- SR4: Design a robot platform that is compact, lightweight and portable.

The most challenging part of throughout this design process is designing a single robotic platform that can fulfill all the system requirements mentioned above. Based on these requirements, SR3 and SR4 are inherent properties of SR1 and SR2. As a result, the achievement of SR1 and SR2 is more than sufficient to satisfy the overall success criteria.

### Step 8: Principles Application & Implementation

Once the system requirements are defined, we analyze the reframed solutions which we extracted from our biological search at the beginning of our process. Then we apply those reframed solutions to create an engineering artifact [i.e., Scorpio (Tan et al., 2016)] that meets the system requirements which satisfy the overall design criteria in the problem definition.

#### Principle Application

A summary of the extracted principles, reframed solutions, and its applications in the development of the Scorpio robot and the connections to the system requirements is presented below:

- *Cebrennus rechenbergi* forms an abstract wheel using its half spherical shaped legs while rolling. This formation helps for an efficient rolling locomotion. Based on this observation, Scorpio's legs are designed to be in half-spherical shape.
- The spider uses eight legs for crawling which gives a stable walk. Based on engineering principles, minimum three legs are required to support a three-dimension object. The spider requires four legs to perform its rolling locomotion due to its spherical formation. Based on this observation, Scorpio will have four legs in total, which are sufficient for stable crawling as well as effective rolling.
- The spider performs an active rolling, which means that it does not require an external force to activate the rolling locomotion. The somersault motion generates a shift in its center of gravity. Similarly, Scorpio uses its legs to push itself to propel from the ground which will shift its center of gravity to achieve the rolling locomotion.
- The somersault performed by the spider makes the whole body to rotate. Based on this, Scorpio's rolling locomotion involves rotating the whole body.
- Primates keep their body closer to the object of interest climbed. This helps to keep the center of gravity close to the object, which can reduce the amount of energy used for climbing. Likewise, Scorpio's wall climbing mechanism design keeps the body as much as close to the wall. This in return reduces the overall energy used throughout the wall-climbing locomotion and thus increases the battery lifetime.
- Scorpio's wall climbing mechanism is a complete adaptation of gorilla's knuckle-walking locomotion along with an additional commercial adhesive (adhesive which uses micro-suction cups to stick to the wall). The gorilla uses its two arms together to struck on the ground while simultaneously pushing forward its body to the direction of motion while performing jumping. The same motion is repeated multiple times to perform wall climbing for Scorpio. The wall-climbing unit involves three pedals where the center pedal is part of the body, and the other two are its arms. A single DC motor coupled with a series of gears and linkage mechanism is used to drive the wall climbing locomotion. This motion can generate the optimal sticking force and the optimal peel of the force required for the stable wall climbing and descending.

#### Implementation

**Figure 7:**
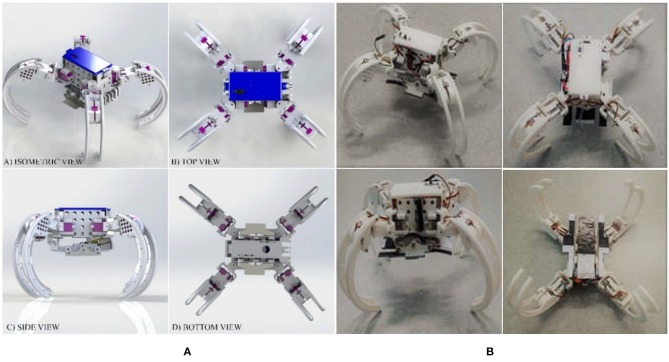
The rendered virtual design **(A)** and physical prototype **(B)** of the Scorpio robot.

**Figure 8:**
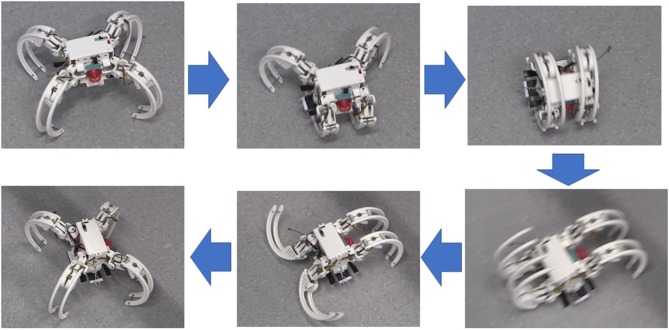
The Scorpio robot performing transformation from crawling to rolling and from rolling to crawling gaits.

Figure 7 demonstrates the design and physical prototype of the Scorpio robot. The robot body is surrounded by four legs named as the tibia, four servo covers named femur, four main joints named as coxa, and a wall climbing mechanism attached underneath. The whole prototype is fabricated through 3D printing with PLA (Poly Lactic Acid) material. The legs are curved inward which helps for a circular formation for rolling. The weight of the legs is reduced by creating structural holes on the surface of each leg. Each leg has a protruded structure which is appropriate for standing. Three servo motors are attached to each leg. This gives 3 DoFs (Degrees of Freedom) to each leg. The crawling motion involves 2 DoFs for each leg, and the crawling-to-rolling transformation involves 3 DoFs for each leg (transformation from crawling to rolling and rolling locomotion gaits are shown in Figure 8). Once the robot transforms into the rolling gait, the legs are responsible for pushing forward the body which shifts the center of gravity of the robot. This trigger is used to achieve the rolling locomotion with 1 DoF.

**Figure 9:**
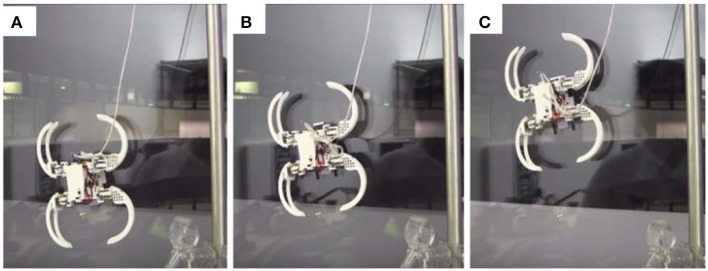
A sequential snapshots **(A–C)** of the wall-climbing scenario of the Scorpio robot.

The wall-climbing module attached underneath the body is composed of three pedals. The mechanism involves a series of gears and linkage mechanisms. A single DC motor is used to drive this mechanism where the motion generated can achieve an optical sticking force and an optimal peel-off force required for a stable wall climbing and wall descending. Figure 9 shows the snapshots of wall climbing performed by the Scorpio robot. Because the focus of this paper is not autonomy, the current version of the robot is controlled manually. The readers are encouraged to refer to Tan et al. (2016) and Yanagida et al. (2017) for details of realization of the Scorpio robot, which are not to reiterate here because they are outside of the scope (i.e., the design process) of this paper.

## Conclusion and Discussion

In this paper, we proposed a novel solution-based process named “System-of-Systems Bio-Inspired Design” process where the inspiration of the engineering product is originated from multiple biological species to solve multiple problems. The goal is to search through available biological solutions in the features of interests, in order to extract principles that can be translated into engineering context. The framework allows multiple features to be considered with multiple categories, which offers a systematic approach in bio-inspired design with intrinsic compliance. Using the proposed SoS BID process, conceptual design and implementation of a reconfigurable robot with multiple locomotion modes were presented and demonstrated. It is shown that the proposed framework provided a step-by-step guideline in developing a robotic platform which can satisfy most of the essential criteria identified in the problem definition, such as navigation through multistory buildings using its multi-modal locomotion capabilities such as crawling, rolling, and wall-climbing. Through different modes of gait, the Scorpio robot was able to achieve stable crawling and rolling locomotion to maneuver in multi-type terrains such as smooth and rough terrains while overcoming obstacles. We believe that the proposed method can simplify the design process, improve the overall efficiency and efficacy, and thus benefit the designers in designing novel bio-inspired robotic platforms with high compliance in terms of the desired features of interests.

It worth pointing out that the proposed framework including eight steps depicts the system-of-systems design process in a generic way, in order to fit in more application cases. The development of the robot in the case study was basically following these eight steps, and of course, the real development process involved some detailed sub-steps in between the eight steps, which are not treated as the skeleton elements of the main framework. These sub-steps might involve specific issues such as material, power, kinematics and dynamics, which should be considered case by case. Here, we are focused on proposing a generic framework which is concise and flexibly open to further add-on treatments. Actually, satisfying both more general and detailed is somehow contradictory. In this paper, we choose the former, namely a general framework, to describe this new design concept and framework for bio-inspired robots, which brings inspiration from multiple species. On the other hand, the most of current available bio-inspired robots in the state of the art are engineering implementations using single species, which adopts different design philosophies from our approach. Thus, if we want to improve the design of such robots in the sense that to integrate more biologically inspired features, the proposed methodology could be used by following the eight steps with certain degree of variation. The key strength of the proposed design process is that it can integrate different inspiration sources. Then, different locomotion modes can be performed by such a unified robot, which outperforms the single-species-inspired robot in versatility of mobility.

The limitation of the proposed framework is 2-fold. Firstly, there are some subjective treatments involved in the implementation of the proposed framework, especially, the selection step of the design process is relatively subjective. This is determined by the nature of the bio-inspired design where different biological sources may lead to similar functionalities. The researchers or designers may select the inspiration sources according to their interests, which is fine as long as the problem can be solved. However, it's hard and somewhat impossible to evaluate all the potential designs with similar functionalities for a specific application (and there is no such work so far) and decide which one is the best compared with others. Secondly, we propose this system-of-systems design framework including a critical step of merging different biological principles. However, how to merge these bio-principles systematically and regularly or handling differences in the requirements when merging different biological principles is a huge theme which need to be explored dedicatedly. Merging different biological principles indeed should be treated case by case, and more importantly, so far, there is no matured principle or guidance on how to merge the principles of different species. We put forward the self-reconfiguration as a powerful merging approach, but haven't studied other alternatives deeply enough yet.

In the future work, we will improve the framework with more strictly structured elements in a more quantitative fashion. In addition, we will investigate more merging methodologies of different biological principles except for the reconfiguration fashion adopted in this paper. Furthermore, the Pareto frontier and multi-objective optimization method will be explored to optimize the design parameters of the robot.

## Data Availability Statement

The raw data supporting the conclusions of this manuscript will be made available by the authors, without undue reservation, to any qualified researcher.

## Author Contributions

NT, NB, and RM designed the study and developed the robot prototype. ZS, SV, RS, and KW provided the guideline and feedback in the structure of the design process. NT and ZS wrote the manuscript in consultation with RM and KW. All authors provided critical feedback and helped shape the research, analysis and manuscript.

### Conflict of Interest

The authors declare that the research was conducted in the absence of any commercial or financial relationships that could be construed as a potential conflict of interest.
